# Development and testing of relative risk-based health messages for electronic cigarette products

**DOI:** 10.1186/s12954-021-00540-1

**Published:** 2021-09-08

**Authors:** Catherine Kimber, Sharon Cox, Daniel Frings, Ian P. Albery, Lynne Dawkins

**Affiliations:** 1 Centre for Addictive Behaviour Research, Division of Psychology, School of Applied Sciences, London South Bank University, 103 Borough Road, London, SE1 0AA UK; 2 Department of Behavioural Science and Health, University College London, Gower Street, London, WC1E 6BT UK

**Keywords:** Electronic cigarettes, E-cigarettes, Health messages, Relative risks messages, Warning labels, Tobacco products directive

## Abstract

**Background:**

Health messages on e-cigarette packs emphasise nicotine addiction or harms using similar wording to warnings on cigarette packs. These may not be appropriate for e-cigarettes which constitute a reduced risk alternative for smokers. This research aimed to (1) develop and test a selection of relative risk messages for e-cigarette products; (2) compare these to the two current EU Tobacco Products Directive (TPD) nicotine addiction messages; and (3) explore differences between smokers, non-smokers and dual users.

**Method:**

Twenty-six messages focusing on either harm-reduction or cessation were developed and rated by multidisciplinary experts for accuracy, persuasiveness and clarity. The eight highest ranking messages were compared alongside the TPD messages in a sample of 983 European residents (316 smokers, 327 non-smokers, 340 dual users) on understandability, believability and convincingness.

**Results:**

On all three constructs combined, the two TPD messages rated the highest, closely followed by four relative risk messages “Completely switching to e-cigarettes lowers your risk of smoking related diseases”, “Use of this product is much less harmful than smoking”, “Completely switching to e-cigarettes is a healthier alternative to smoking”, and “This product presents substantially lower risks to health than cigarettes” which did not differ statistically from the TPD messages. Non-smokers rated TPD1 significantly higher overall than dual users. Dual users rated “This product is a safer alternative to smoking” significantly higher than non-smokers. Messages did not differ on understandability.

**Conclusions:**

These alternative messages provide a useful resource for future research and for policy makers considering updating e-cigarette product labelling.

**Supplementary Information:**

The online version contains supplementary material available at 10.1186/s12954-021-00540-1.

## Background

Health warnings can increase awareness about the dangers of smoking and promote cessation [1, 2] and have become central to anti-smoking education campaigns worldwide. In addition to reducing smokers’ desire for tobacco cigarettes [3, 4], health warnings have the potential to prevent initiation in non-smokers [5]. Similar labels are now displayed on electronic cigarette (EC) packs, usually with a focus on addictiveness. These messages are often expressed in absolute terms and do not contain comparative harm information which may deter smokers from moving to a relative risk product [6]. Indeed, “switching one addiction for another” is one of the key cited reasons for not trying an EC and perceptions of their risk relative to smoking is increasing [7]. A more nuanced set of health messages may be advantageous. The current study describes a comprehensive methodology used to develop and assess (in terms of understandability, believability and convincingness) a set of messages which highlight the relative risks of EC compared to tobacco smoking.

Since their introduction, uptake and awareness of EC has increased [8, 9]; current use is estimated at 3.2 million adults in Great Britain [10]. Smoking cessation and reduction remain the most commonly cited reasons for use, and there is increasing evidence for their role in supporting smoking abstinence [11–14]. However, initiating or increasing EC use whilst cigarette smoking is maintained is unlikely to lead to substantial improved health outcomes [15]. Despite the concerns vis-à-vis the increasing prevalence rates amongst youth [16, 17] and potential health harms specifically for users who continue to smoke concurrently [18], findings from emission, biomarker and switching studies suggest that EC are considerably less harmful than tobacco cigarettes [15, 19–27], a conclusion endorsed by public health agency reviews [28, 29].

More recently, public misperceptions of harms associated with EC and nicotine use have increased. For example, only 17% of UK respondents (*N* = 12,070) have been shown to correctly believe that EC are considerably less harmful than tobacco smoking [30]. Similarly, 32.2% of former and current smokers (*N* = 1720) in the UK reported that substituting tobacco cigarettes for e-cigarettes reduced harm to health [31]. These misperceptions extend to EC addictiveness compared to smoking with only 25.4% perceiving the former as less addictive [31]. Despite widespread public health endorsement of EC in England such misperceptions were commonplace [32]. These harm misperceptions extend to other countries; only 5% of Greeks perceived EC to be less harmful than cigarettes [33]; 54.7% of a US sample reporting EC to be not at all to moderately harmful [34]; and longitudinal evidence in the US suggesting a twofold increase in beliefs that EC are more or equally as harmful to health than tobacco cigarettes [35].

One likely contributory factor for such misperceptions may be health warnings on EC packs. In the EU, the current regulatory framework stipulates that all EC packets and refill products must carry a nicotine health warning (“*This product contains nicotine which is a highly addictive substance*” or “*This product contains nicotine which is a highly addictive substance. It is not recommended for non-smokers*”) [36], which may inflate perceptions of harm and addictiveness in readers. In support of this, studies have found the presence of a nicotine warning in an EC advertisement to increase risk beliefs, harm perceptions and addictiveness [37], and dissuade use intentions [38]. Similarly, in a UK sample of daily smokers (*N* = 95), we found that exposure to the EU-TPD (EU Tobacco Product Directive) message reduced EC use and purchase intentions [6]; in a larger trial (*N* = 2495 UK residents) increased both smokers’ and non-smokers’ harm perceptions [39]. Similarly, focus groups with EC users and smokers in the US suggest that health warnings deemed too negative may have the unintended consequence of reducing appeal among smokers who may be considering EC for smoking cessation [40].

Not all messaging has such effects; research suggests that *advertising* messages that have focused on differences between cigarettes and EC (e.g. helps to quit smoking) rather than similarities (feels like smoking, relieves cravings) created more interest among smokers in trying an EC [41]. Moreover, whilst advertisements containing warnings increase addiction related risk beliefs, the concurrent presence of a EC health message (i.e. a comparative risk message) nullifies this effect [42].

One way to dispel erroneous perceptions and encourage use of EC in smokers may be to refocus warning labels away from *absolute* potential harms of EC to ones conveying risks *relative* to smoking. Whilst there is a clear rationale for developing such messages and conducting initial evaluations of their effectiveness, no such work has been undertaken. This study aimed to (1) develop a set of relative risk health messages which convey the relative risks of EC compared to tobacco cigarettes that are believable, understandable and convincing; (2) compare these messages to the existing TPD nicotine addiction warnings currently implemented in the UK and in other EU countries; and (3) explore differences between smokers, non-smokers and dual users in how believable, understandable and convincing they rated the messages.

## Methods

### Initial message development

Drawing on our subject-specific knowledge of EC and with reference to (1) the general guiding principles outlined in the health communication message review tool (National Collaborating Centre for Methods and Tools [43]), and (2) specific recommendations for the development of tobacco cigarette health warnings (i.e. the European Commission report [44], Institute of Global Tobacco Control [45] and the Eurobarometer Aggregate Report [46]), initial messages were developed by the authors with a focus on the relative risk of EC vs. smoking, and the benefits of completely switching to vaping. Given the focus here was to generate messages which encourage smokers to switch to a less harmful product rather than to develop warning messages to deter smokers, the literature on cigarette health warnings was less relevant. However, it was used here to inform the development of messages in terms of possible presentation parameters (i.e. content, length, prominence on pack) of health messages that may influence the extent to which they will be noticed and acted upon. It is also important that health messages are clear, comprehensive and credible so as to increase level of attention and likelihood of recall [43], including for individuals with low literacy [5]. Twenty-six initial messages were developed incorporating a range of grammatical (second [“you” or “your”] and third [“they” or “smokers”]) person perspectives to account for the variability in which individuals attend to, process and respond to mass communication messages [47, 48]. These messages were also developed using specific versus general risk and exposure information (e. g. “*Switching to e-cigarettes reduces damage to your lungs”* vs. “*Switching to e-cigarettes reduces your health risks”* and “*E-cigarettes reduce harmful toxin exposure to those around you”*).

### Consultation and refinement phase

To communicate health messages effectively, it is important that messages are accurate and clear (free of technical terms and jargon) to ease readability and minimise cognitive effort required to engage with the materials [49]. One way to verify information accuracy, clarity of the wording and tailoring to lay audiences is to refer to academic knowledge and expertise. Thus, in a preliminary phase, we approached 12 experts (senior academics, behavioural scientists, health psychologists, policy advisors and experienced vapers; see acknowledgements section) in the e-cigarette, tobacco control and behaviour change field. Of these, 8 agreed to rate the 26 messages on accuracy, persuasiveness and clarity, each on a 5-point scale (0—not at all to 5—extremely). These experts were also asked to select the 3 messages that they would recommend and 3 messages that they would abandon. One ranking system was created for each of the reviewers for scores on accuracy, persuasiveness and clarity then averaged and Friedman tests applied to identify the most highly rated, and the most and least recommended.

In the latter phase, the eight messages that scored the highest and were not advised by the experts to be abandoned were selected for further evaluation by the general public and rated on understandability, convincingness and believability, constructs that are likely to contribute to generating favourable engagement of the target audience, increase persuasiveness of the health messages [49], and in turn drive behaviour change.

**Fig. 1:**
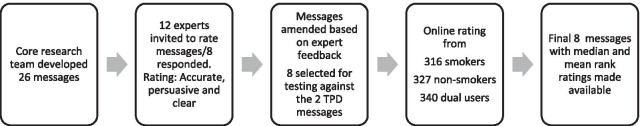
Method of development and testing of relative risk health messages for electronic cigarette packs

Figure 1 illustrates the method used to develop and test the health messages. On the recommendation of two experts, messages were amended to add the word “completely” to precede the word “switching” (see Table 1). The list of 26 relative risk messages is presented in Table 1 (selected messages are emboldened).

**Table 1:** Median and Mean rank for the Initial Health messages on all 3 constructs combined Accuracy, Persuasiveness and Clarity

Relative risk health messages	Percentiles 50th median [25th-75th]	Mean rank
RRM1. [EC]^a^ are likely to be 95% less harmful than smoking	3.83 [2.58–4.67]	13.83
RRM2. [EC] reduce smoking related health risks by 95%	3.83 [2.83–4.08]	11.33
RRM3. Smoking is 95% more harmful than vaping	2.5 [1.58–3.67]	3.92
RRM4. No product is completely safe but use of this product is much less harmful than smoking	3.67 [3–4.17]	12.92
**RRM5. Use of this product is much less harmful than smoking**^**1**^	4.17 [3–4.75]	**16.92**
RRM6. No tobacco related product is safe but this product presents substantially lower risks to health than cigarettes	3.33 [3–3.75]	9.58
**RRM7. This product presents substantially lower risks to health than cigarettes**^**5**^	4.17 [3.58–4.33]	**18.08**
**RRM8. [Completely] switching to [EC] reduces your health risks**^**2**^	4 [3.5–4.33]	**15.42**
**RRM9. [Completely] switching to [EC] reduces your cancer risk**	3.83 [3.5–4.42]	**15.33**
RRM10. Switching to e-cigarettes improves your health	3.17 [2.5–3.67]	5
**RRM11. [Completely] switching to [EC] lowers your risk of smoking related diseases**^**6**^	4.17 [3.67–4.33]	**17.42**
**RRM12. This product is a safer alternative to smoking**^**3**^	4.33 [3.33–5]	**20.33**
RRM13. This product is a safer alternative for smokers	4.33 [3.33–5]	20.33
RRM14. Switching to [EC] reduces damage to your lungs	3.33 [3–4.5]	13.67
RRM15. Switching to [EC] reduces your chances of developing cancer	3.83 [3.58–4]	12.92
RRM16. [EC] contain no tar, the toxic component of smoke	4 [3.42–4.33]	14.92
RRM17. [EC] contain far fewer toxins than tobacco cigarettes	3.67 [3.5–4.33]	13.17
RRM18. [EC] reduce harmful toxin exposure to those around you	3.67 [3.33–3.75]	12.17
RRM19. [EC] reduce exposure to second-hand smoke	3.67 [3–4]	11.75
**RRM20 Completely switching to [EC] is a healthier alternative to smoking**^**7**^	3.5 [2.5–4.42]	**19.75**
RRM21 It is recommended that smokers should switch to [EC] ^8^	3.5 [2.5–4.42]	11.08
RRM22 Many smokers who switch to e-cigarettes report improved health^8^	3.67 [2.92–4.17]	12.75
RRM23 Like other smokers, if you switch to e-cigarettes you will experience improved health	3.67 [2–4]	9
RRM24 Using an e-cigarette improves your chances of quitting^4^	3.83 [3.25–4.33]	14.33
**RRM25. Using an [EC] doubles your chances of quitting smoking**^**8**^	3.33 [3.17–4.17]	**11.50**
RRM26. Using an [EC] reduces cravings for cigarettes	3.67 [3.25–4.08]	13.58

^a^[EC] e-cigarettes featured in full; Bold font indicate selected messages (*n* = 8) further evaluated in this second phase study (*N* = 983); Friedman test x^2^(25, *n* = 6) = 44.02, *p* = .011 found a significant difference on all 3 constructs combined; Friedman test x^2^(25, *n* = 8) = 43.89 *p* = .011 also revealed the top 8 most recommended messages indicated by lower case superscript numbered 1–8; For messages to abandon, Friedman test x^2^(25, *n* = 8) = 35.14, *p* = .086 found not significant differences but individual experts’ comments and advice on messages to “abandon” vis-à-vis the complexity and length of messages were taken into consideration during the selection process

### Evaluation of messages by the general public

#### Participants and design

Participants were recruited online, largely via *Figure Eight* (an online crowd-labour platform), social media sites (Facebook) and a student-focused Research Participation Scheme (RPS) at London South Bank University (LSBU). All measurement materials were hosted and delivered by Qualtrics between September and October 2018. Payment was US$2.00 (~ €0.89) per participant taking part by Figure Eight or course credit for student participants. Of the 1,148 participants who consented to take part, those who failed to report their smoking/vaping status (*n* = 140) and those who reported as exclusive EC users (*n* = 25) were excluded from all data analyses as they were not part of the main target sample and were not suitable for further sub-group analyses due to small cell counts. The final sample of 983 participants comprised 316 smokers, 327 non-smokers, 340 dual users. Inclusion criteria for the study were: aged 18 + , resident in Europe and fluent in English. Participants were randomly assigned to view only *one* of the messages resulting in between 90 and 117 ratings per message. A post hoc achieved power analysis revealed the sample was sufficient to detect (in a one-way ANOVA design with 10 conditions, see Analysis below) a medium effect size (f = 0.25) with an alpha of 0.05 at power = 0.99.

#### Materials

Eight relative risk messages (Table 1) plus two TPD warnings, TPD1 “*This product contains nicotine which is a highly addictive substance*” and TPD2 “*This product contains nicotine which is a highly addictive substance. It is not recommended for non-smokers*” received ratings on understandability, believability and convincingness. These dimensions have been used previously to evaluate the efficacy of smoking cessation media messages [50] and EC warning statements [51]. All messages were displayed at the centre of the screen in black colour and Helvetica font bold type occupying 30% of the screen on a white background.

#### Measures

*Baseline measures* Demographic variables were gender, age, ethnicity, occupation, economic activity and highest attained qualification (consistent with data collected by the UK Office for National Statistics). Occupation was measured with a single item comprising four categories: “routine and manual”, “intermediate”, “managerial and professional occupation”, and “never worked & long-term unemployed” [52].

Smoking status was recorded as “*non-smoker*”, “*daily*” and “*occasional smoker*”. EC use status was collected by asking: “*Do you currently use an EC?”* Response options were: “*Yes, daily*”, “*Yes, occasionally*”, “*I have never used one*”, “*I used one occasionally (not daily) in the past and no longer use it*”, and *“I used one every day in the past and no longer use it”*. For smokers, motivation to quit was measured using the Motivation to Stop Scale (MTSS), a single-item measure of intention, desire to stop smoking and the immediacy of quit date intention [53]. Cigarette dependence was measured using the Fagerström Test for Cigarette Dependence (FTCD) [54].

#### Primary outcomes

All ten messages were rated for understandability, believability and convincingness by asking *how [believable – understandable—convincing] the health message was*. The response options were on Likert type scales (response options “*Extremely* = *7, Very* = *6, Moderately* = *5, Neutral* = *4, Somewhat* = *3, Slightly* = *2 and Not at all* = 1”). Prior to and following the experimental exposure to one of the 10 EC health messages, perceptions of harmfulness, addictiveness and social acceptability were measured, and participants were also asked to recall the health message (data to be reported separately).

### Statistical analysis

There were no significant associations between participant demographics and assigned message type (all *p*s > 0.09). Descriptive statistics (mean, SD) for each message relating to ratings of understandability, believability, convincingness and the total score were then obtained. The Cronbach alpha coefficient for the total (aggregate) score was 0.74. To explore differences in understandability, believability, convincingness and the aggregate score between messages (10 levels) and smoking status (exclusive smokers vs. non-smokers vs. dual users [concurrent smokers & EC users]), a two-way between-subjects ANOVA was conducted. As homogeneity of variances assumption was violated (Levene’s test *p* < 0.05), we conducted this analysis with 1000 bootstrap samples [55]. Significant main effects of message and smoking status were explored using Bonferroni post hoc tests and significant interactions were followed up using simple effects analyses. The analysis was not pre-registered and the results should be considered exploratory.

## Results

Table 2 summarises participant demographics and smoking characteristics.

**Table 2:** Participants’ Characteristics

	*N* = 983	%	Mean	SD	Min	Max
*Gender*	**–**	**–**	**–**	**–**	**–**	**–**
Male	635	61.7	**–**	**–**	**–**	**–**
Female	393	38.2	**–**	**–**	**–**	**–**
Non-binary/prefer not to disclose	2	0.2	**–**	**–**	**–**	**–**
*Ethnicity*	**–**	**–**	**–**	**–**	**–**	**–**
White	998	96.9	**–**	**–**	**–**	**–**
Black/African/Caribbean	6	.6	**–**	**–**	**–**	**–**
Mixed/Multiple ethnic background	13	1.3	**–**	**–**	**–**	**–**
Asian (incl. *n* = 1 Other)	13	1.3	**–**	**–**	**–**	**–**
*Countries of origin*^1^	**–**	**–**	**–**	**–**	**–**	**–**
United Kingdom	142	13.8	**–**	**–**	**–**	**–**
Ukraine	145	14.1	**–**	**–**	**–**	**–**
Spain	94	9.1	**–**	**–**	**–**	**–**
Italy	90	8.7	**–**	**–**	**–**	**–**
Poland	61	5.9	**–**	**–**	**–**	**–**
Bosnia & Herzegovina	52	5.0	**–**	**–**	**–**	**–**
Germany	46	4.5	**–**	**–**	**–**	**–**
Romania	46	4.5	**–**	**–**	**–**	**–**
Other European countries	278	26.96				
*Occupation*	**–**	**–**	**–**	**–**	**–**	**–**
Routine and manual	234	22.7	**–**	**–**	**–**	**–**
Intermediate	257	25	**–**	**–**	**–**	**–**
Managerial & professional	433	42	**–**	**–**	**–**	**–**
Never worked & Long term unemployed	106	10.3	**–**	**–**	**–**	**–**
*Economic activity*	**–**	**–**	**–**	**–**	**–**	**–**
Employed (incl. employed & studying)	785	76.2	**–**	**–**	**–**	**–**
Unemployed & looking for work	96	9.3	**–**	**–**	**–**	**–**
Unemployed & studying	50	4.9	**–**	**–**	**–**	**–**
Retired	30	2.9	**–**	**–**	**–**	**–**
Never worked &/or long term unemployed (incl. home carer)	69	6.7	**–**	**–**	**–**	**–**
*Highest qualification to date*	**–**	**–**	**–**	**–**	**–**	**–**
Degree (or equivalent)	321	31.3	**–**	**–**	**–**	**–**
Higher education (below degree level)	386	37.4	**–**	**–**	**–**	**–**
A-levels or highers	129	12.5	**–**	**–**	**–**	**–**
ONC or National level BTEC	22	2.1	**–**	**–**	**–**	**–**
*Age*	**–**	**–**	37.81	10.60	18	83
*Smoking/Vaping status*^2^	**–**	**–**	**–**	**–**	**–**	**–**
Current exclusive smokers	316	30.68	**–**	**–**	**–**	**–**
Current Dual users	340	33.00	**–**	**–**	**–**	**–**
Non-smokers/Non-vapers	327	31.75	**–**	**–**	**–**	**–**
*FTCD*^*3*^ (*N* = 660)	**–**	**–**	3.81	2.45	0	9
*MTSS*^*4*^* (N* = *660)*	**–**	**–**	3.44	1.55	1	7

^1^**Countries of origin** excludes missing data (*n* = 25) and does not sum to 100% of the sample. Only those with a frequency greater than *n* = 40 is shown, see Additional file 4: Table S1 for a more detailed list of countries; ^2^**Smoking/Vaping status** includes daily and occasional current use, 22 cases were missing; ^3^**FTCD** = Fagerström Test for Cigarette Dependence; ^4^**MTSS** = Motivation to Stop [Smoking] Scale, both were measured in smokers only

### Aggregate ratings across understandability, believability and convincingness.

**Fig. 2:**
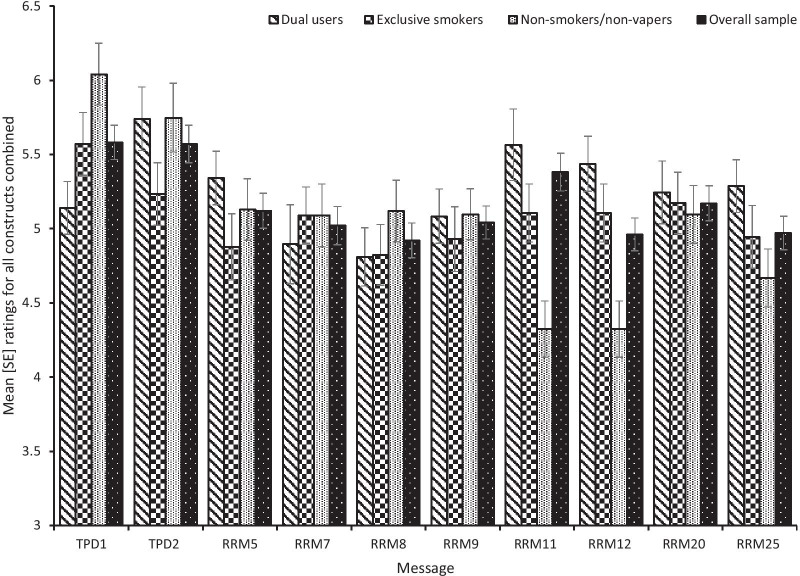
Overall mean (SE) ratings across all constructs combined per group and for the overall sample

A main effect of message, *F*(9, 953) = 4.49, *p* = 0.001, *ƞ*_*p*_^*2*^ = 0.041, was shown. TPD1, TPD2, and relative risk messages (RRM) 11, 5, 20 and 7 did not differ significantly (all ps > 0.05). TPD1 and TPD2 scored significantly higher than RRM12, RRM8 and RRM25, whilst RRM9 scored significantly lower than TPD1 but not TPD2 (see Table 3 for mean [SD] ratings for the overall sample and Fig. 2 for mean [SE] ratings per smoking status group). No main effect of smoking/vaping status was found, *F*(2, 953) = 1.32, *p* = 0.268, *ƞ*_*p*_^*2*^ = 0.003. A significant message by smoking status interaction was shown, *F*(18, 953) = 2.33, *p* = 0.001, *ƞ*_*p*_^2^ = 0.042. Non-smokers attributed higher scores to TPD1 compared to dual users (*M* = 6.04, 95% CI [5.66–6.42] vs. *M* = 5.14, 95% CI [4.72–5.56]) *p* = 0.006), whilst dual users rated RRM12 *“This product is a safer alternative to smoking”* more favourably than non-smokers (*M* = 5.44, 95% CI [5.10–5.77] vs. *M* = 4.32, 95% CI [3.92–4.73]) *p* < 0.001).

**Table 3:** Mean (SD) per message for ratings on each subscale and the aggregate ratings

Messages	Understandability	Believability	Convincingness	Overall scales*
TPD1. *This product contains nicotine which is a highly addictive substance* (*n* = 103)	5.51 (1.76)	5.57 (1.43)^A^	5.52 (1.40)^A^	5.54 (1.24)^ACDEF^
TPD2. *This product contains nicotine which is a highly addictive substance. It is not recommended for non-smokers* (*n* = 84)	5.83 (1.48)	5.51 (1.50)^B^	5.33 (1.48)^B^	5.56 (1.38)^BCDF^
RRM5. *Use of this product is much less harmful than smoking* (*n* = 99)	5.49 (1.64)	5.06 (1.38)	4.89 (1.39)^a^	5.15 (1.13)
RRM7. *This product presents substantially lower risks to health than cigarettes* (*n* = 86)	5.43 (1.41)	4.90 (1.24)^a^	4.81 (1.40)^a^	5.09 (1.15)
RRM8. *Completely switching to e-cigarettes reduces your health risks* (*n* = 98)	5.45 (1.56)	4.71 (1.46)^ab^	4.57 (1.46)^ab^	4.91 (1.16)^abd^
RRM9. *Completely switching to e-cigarettes reduces your cancer risk* (*n* = 113)	5.71 (1.43)	4.76 (1.54)^ab^	4.68 (1.55)^a^	5.05 (1.21)^ae^
RRM11. *Completely switching to e-cigarettes lowers your risk of smoking related diseases* (*n* = 86)	5.85 (1.20)	5.17 (1.27)^C^	5.07 (1.26)	5.36 (1.05)
RRM12. *This product is a safer alternative to smoking* (*n* = 111)	5.56 (1.59)	4.76 (1.44)^ab^	4.57 (1.42)^ab^	4.96 (1.17)^abc^
RRM20. *Completely switching to e-cigarettes is a healthier alternative to smoking*				
(*n* = 96)	5.64 (1.32)	5.01 (1.17)	4.85 (1.26)^a^	5.17 (.96)
RRM25. *Using an e-cigarette doubles your chances of quitting smoking*				
(*n* = 107)	5.69 (1.33)	4.54 (1.71)^abc^	4.73 (1.53)^a^	4.99 (1.23)^abf^

^*^Mean (SD) for the overall scales correspond to the aggregate ratings across understandability, believability and convincingness combined

Messages sharing a superscript letter denote significant differences (*p* < .05) where upper case (capital) letters indicate significantly higher rating vs. their corresponding lower-case letters

For example, for the main effect of message [*F*(9, 953) = 4.493, *p* = .001, *ƞ*_*p*_^2^ = .041], for all subscales combined, A indicates TPD1 scored significantly higher compared to RRM, 8, 9, 12 and RRM25; B indicates that TPD2 scored significantly higher than RRM, 8, 12 and 25; C indicates TPD1 and 2 scored significantly higher than RRM12; D indicates TPD 1 and 2 scored significantly higher than RRM8; E indicates TPD1 scored significantly higher than RRM9; *F* indicates TPD1 and 2 scored significantly higher than RRM25

### Message understandability ratings

There were no main effect of message, [*F*(9, 953) = 0.88, *p* = 0.54)], smoking status [*F*(2, 953) = 0.81, *p* = 0.44] or message by smoking status interaction [*F*(18, 953) = 1.17, *p* = 0.28] for understandability (see Table 3 for mean (SD) and Additional file 1 for means [SE]).

### Message believability ratings

Main effects for message [*F*(9, 953) = 6.16, *p* < 0.001, *ƞ*_*p*_^2^ = 0.055)], and smoking/vaping status [*F*(2, 953) = 3.16, *p* = 0.04, *ƞ*_*p*_^2^ = 0.007] were found. Mean believability scores for TPD1 and TPD2 were highest followed by RRM11 “*Completely switching to e-cigarettes lowers your risk of smoking related disease”, RRM5 “Use of this product is much less harmful than smoking*” *and RRM20 “Completely switching to e-cigarettes is a healthier alternative to smoking*”; these 5 messages did not differ statistically from each other (all *p*s > 0.05)*.* TPD1 was rated as the most believable and differed significantly from RRM7 (*p* = 0.028), RRM8 (*p* < 0.001), RRM9 (*p* = 0.001), RRM12 (*p* = 0.001) and RRM25 *(p* < 0.001). TPD2 rated second most believable and differing significantly from RRM8 (*p* = 0.005), RRM9 (*p* = 0.008), RRM12 (*p* = 0.006) and RRM25 (*p* < 0.001). See Table 3 for mean (SD) and Additional file 2 for mean [SE] ratings.

A significant message by smoking/vaping status interaction [*F*(18, 953) = 1.78, *p* = 0.023, *ƞ*_*p*_^*2*^ = 0.033] was shown. Dual users rated RRM12, *“This product is a safer alternative to smoking”,* as more believable (*M* = 5.33, 95% CI [4.90–5.76] compared to non-smokers (*M* = 4.11, 95% CI [3.57–4.65], *p* = 0.001). Smokers rated RRM5, *“Use of this product is much less harmful than smoking*”, significantly less believable compared to dual users (*M* = 4.52, 95% CI [3.98–5.05] vs. *M* = 5.32, 95% CI [4.88–5.75], *p* = 0.05).

### Message convincingness ratings

Significant main effects for message [*F*(9, 953) = 5.75, *p* < 0.001, *ƞ*_*p*_^*2*^ = 0.052], and smoking/vaping status [*F*(2, 953) = 3.76, *p* = 0.02, *ƞ*_*p*_^*2*^ = 0.008] were shown. The mean score for TPD1 (*M* = 5.52, 95% CI [5.24–5.79]) was the highest followed by TPD2 (*M* = 5.33, 95% CI [5.01–5.64]) then RRM11 *“Completely switching to e-cigarettes lowers your risk of smoking related diseases”* (*M* = 5.07, 95% CI [4.79–5.35]) and RRM5 *“Use of this product is much less harmful than smoking”* (*M* = 4.89, 95% CI [4.61–5.17])*.* TPD1 rated as significantly more convincing than all other messages (all *p* < 0.05) except from TPD2, and RRM11. RRM11 in turn, was rated as significantly more convincing than RRM8, RRM9, RRM12 and RRM25 (*p* < 0.05). See Table 3 for mean (SD) and Additional file 3 for mean (SE) ratings.

A significant message by smoking/vaping status interaction [*F*(18, 953) = 2.49, *p* = 0.001, *ƞ*_*p*_^*2*^ = 0.045] was shown. Non-smokers rated TPD1 as more convincing and RRM25,* “Using an e-cigarette doubles your chances of quitting smoking”,* as less convincing compared to dual users (TPD1: *M* = 5.00, 95% CI [4.52–5.48] vs. *M* = 6.16, 95% CI [5.78–6.54], *p* = 0.001; RRM25: *M* = 4.20, 95% CI [4.08–5.05] vs. *M* = 5.29, 95% CI [4.92–5.65], *p* = 0.005). Non-smokers rated RRM12, *“This product is a safer alternative to smoking”,* as less convincing than dual users (*M* = 3.78, 95% CI [3.29–4.258] vs. *M* = 5.21, 95% CI [4.82–5.59], *p* < 0.001) and smokers (*M* = 4.69, 95% CI [4.27–5.10], *p* = 0.012).

## Discussion

Current health warnings, such as those implemented by the TPD, may inadvertently deter smokers from initiating EC use and substituting their smoking for vaping due to their sole emphasis on the potential health-related harms of nicotine [6, 32]. This may be problematic to the extent that, for smokers, EC have been shown to be more efficacious than nicotine replacement therapies [13] as well as useful tools to prevent relapse [56]. One way to encourage smokers to use EC to promote a switch away from smoking may be to refocus warning labels away from absolute potential harms to ones conveying risks relative to smoking. In this paper, we describe the development of a set of eight alternative health messages for EC packages and the testing of these messages against the current TPD warning messages on understandability, believability and convincingness. We also present differences between smokers, non-smokers and dual users.

From 26 original messages, we explored perceived understandability, believability and convincingness of the 8 relative risks messages most highly rated by experts and 2 current TPD messages. The two current TPD messages were consistently rated the highest for combined understandability, believability and convincingness rating, but did not differ significantly from four relative risk messages; “*Completely switching to e-cigarettes lowers your risk of smoking related diseases”* (RRM11), *“Use of this product is much less harmful than smoking”* (RRM5), “*Completely switching to e-cigarettes is a healthier alternative to smoking”* (RRM20), and “*This product presents substantially lower risks to health than cigarettes”* (RRM7)*.* In terms of the distinct dimension ratings, all messages were rated highly on “understandable”. Importantly, we did not detect a difference between relative risk messages and the TPD messages in this domain; mean ratings were all above 5 indicating that they were as understandable as the current TPD standard. The messages that scored the highest on “believable” and “convincing” were RRM11 and RRM5 alongside the two TPD messages. Compared to dual users, non-smokers attributed higher scores to TPD1 (which emphasises nicotine addiction), whilst dual users rated RRM12, *“This product is a safer alternative to smoking”,* more favourably than non-smokers.

What may explain the observation that the TPD received the highest ratings compared to the relative risk messages overall? One possibility regarding believability may be due to pre-existing beliefs around the health risks and addictive properties of nicotine. There are many examples in the literature demonstrating public misperceptions of the harms of nicotine [32]. In one online survey, smokers reported beliefs that very low nicotine cigarettes were less carcinogenic [57] whilst only 8.6% of a UK sample had accurate harm perceptions, that is, a very small amount of the harm of smoking comes from nicotine [58]. Our own recent work has shown TPD messages were associated with greater addictiveness and harm perceptions of EC especially in non-smokers/non-EC users [39]. Hence, the nicotine addiction warning conveyed by the TPD may align with current tobacco and nicotine beliefs.

Another explanation for the higher endorsement of the TPD as the most believable and convincing could be due to previous exposure. It is plausible that through repeated exposure, this familiarity may enhance message credibility and acceptability [59]. It is highly encouraging that despite the lack of familiarity with the relative risk messages, four messages did not statistically differ from the TPD messages. However, this also means that a familiarity explanation is not sufficient in isolation. Indeed, it could be argued that because novelty requires greater cognitive demand, new, unfamiliar content may increase attention to these messages. Regardless of preferred explanation, that these messages were rated as favourably as the TPD in a sizeable European sample, suggests that such relative risk messages have equal persuasive potential and good utility for future studies exploring how best to communicate the relative risks of EC on EC packs.

Importantly, our data did not indicate that these relative risk messages would lead to unintended consequences in non-smokers. Consistent with our previous findings [39] and those of others [31, 60, 61], non-smokers were more likely to endorse the TPD messages. More specifically, non-smokers ascribed greater believability and convincingness to the TPD1 compared to dual users, whilst dual users tended to rate the relative risk messages (i.e. “*This product is a safer alternative to smoking*”) more favourably in comparison to non-smokers and (i.e. *“Use of this product is much less harmful than smoking”*) compared to smokers. That non-smokers were less likely to endorse the relative risks messages compared to dual users may possibly be an artefact of the study given that these were not government-mandated warning labels which may have had an impact on their credibility. However, importantly this suggests that non-smokers may be less receptive to such messages. This is important insomuch that persuasive health messaging on EC packaging should not deter smokers to switch to reduced risk products whilst not enticing non-smokers. One possible explanation for the higher ratings of the relative risk messages in dual users compared to smokers may be that they are better informed about EC relative risks due to their experience of using the product [62]; or it is also possible that because they are invested and involved, they may have been more attentive to these messages thereby more likely to endorse them [63]. Thus, ways of enhancing the credibility of relative risk messages certainly warrants further investigation. Because such relative risk messages (“*Use of this product is much less harmful than smoking*” RRM5) are more likely to increase use intentions in smokers than non-smokers [39], they hold promise for further empirical investigation.

Whilst the current paper focuses on the development of the messages, it does not determine the extent to which these messages correct misperceptions and increase knowledge around harms of nicotine and EC; this could be the focus for future research. It would be useful to evaluate the universality of these messages by testing them in different regulatory environments outside of the EU as well as in developing countries with high smoking prevalence. Future work could also evaluate these relative risk messages’ persuasiveness potential further by testing their legibility (fonts, colour, font size, positioning and prominence on the pack, background contrast, and so on), readability, memorability and perceptual fluency (how easily and favourably they can be processed and evaluated).

The method used here presents some potential limitations. Although a reasonable sample size was used, a little above 10% were based in the UK and the vast majority were from other European countries. It is, therefore, possible that the results were influenced by differential past exposure and familiarity with the TPD messages. It is worth noting that our analyses did not include exclusive EC users. Given dual users showed greater endorsement of the relative risk messages compared to smokers, these ratings could have been even higher had exclusive EC users been included. A further possible limitation is the over-representation of individuals from managerial and professional groups over those from routine and manual occupations given that smoking is largely concentrated in the latter as opposed to the former groups. Whilst useful, findings are confined to intentions and do not explore changes or fluctuations in perceptions or, how intentions translate into behaviours.

## Conclusions

Here, we present the phased method that allowed the development of eight messages initially selected from a series of 26 rated by a panel of experts and thereafter, rated by the general public in a European sample. Findings from this study are of relevance for public health as they may benefit the understanding of how best to communicate relative health risks associated with EC. These relative risk messages are intended for use by regulators and policy-makers as alternative messages to current heath messages on EC packs. We encourage other researchers to further explore their usefulness and effects on perceptions and/or intentions in different populations and settings.

## Supplementary Information

**Additional file 1: Fig. S1**. Mean (SE) ratings for understandability for dual users, smokers, non-smokers and the overall sample.

**Additional file 2: Fig. S2**. Mean (SE) ratings for believability for dual users, smokers, non-smokers and the overall sample.

**Additional file 3: Fig. S3**. Mean (SE) ratings for convincingness for dual users, smokers, non-smokers and the overall sample.

**Additional file 4: Table S1**. Detailed list of countries.

## Data Availability

The datasets used and analysed during the current study are available from the corresponding author on reasonable request.

## Abbreviations

EC: E-cigarettes
MTSS: Motivation to Stop Smoking
EU-TPD: European Union Tobacco Products Directive
FTCD: Fagerström Test of Cigarette Dependence
RRM: Relative Risk Messages
