# Supplementary material for: Development and testing of relative risk-based health messages for electronic cigarette products
Source: Harm Reduct J. 2021 Sep 8;18:96. doi: 10.1186/s12954-021-00540-1 (PMC8424813; doi:10.1186/s12954-021-00540-1)
Supplement: Supplementary file 3 — Additional file 3: Fig. S3. Mean (SE) ratings for convincingness for dual users, smokers, non-smokers and the overall sample. [file 12954_2021_540_MOESM3_ESM.docx]

**Figure S3** Mean (SE) ratings for convincing for dual users, smokers, non-smokers and the overall sample
