# Supplementary material for: Development and testing of relative risk-based health messages for electronic cigarette products
Source: Harm Reduct J. 2021 Sep 8;18:96. doi: 10.1186/s12954-021-00540-1 (PMC8424813; doi:10.1186/s12954-021-00540-1)
Supplement: Supplementary file 4 — Additional file 4: Table S1. Detailed list of countries. [file 12954_2021_540_MOESM4_ESM.docx]

| **Table S1** Detailed list of countries | | | | |
| --- | --- | --- | --- | --- |
|  | **N=983** |  | **%** | |
| **Countries of origin**^1^ | **-** | **-** | |  |
| United Kingdom | 142 | 13.8 | |  |
| Ukraine | 145 | 14.1 | |  |
| Spain | 94 | 9.1 | |  |
| Italy | 90 | 8.7 | |  |
| Poland | 61 | 5.9 | |  |
| Bosnia & Herzegovina | 52 | 5.0 | |  |
| Germany | 46 | 4.5 | |  |
| Romania | 46 | 4.5 | |  |
| Bulgaria | 38 | 3.7 | |  |
| Portugal | 37 | 3.6 | |  |
| Macedonia | 33 | 3.2 | |  |
| Greece | 31 | 3.0 | |  |
| France | 23 | 2.2 | |  |
| Croatia | 22 | 2.1 | |  |
| Austria | 13 | 1.3 | |  |
| Other European countries^a^ | 81 | 7.86 | |  |
| *Note*. ^1^***Countries of origin*** *excludes missing data (n = 25) thus does not add up to 100% of the sample, only those with a frequency of above n = 10 is shown and* ***Other European countries*** *includes those with frequency ranging from n = 1 to 9* | | | | |
